# Use of precision-fed cecectomized rooster assay and digestible indispensable amino acid scores to characterize plant- and yeast-concentrated proteins for inclusion in canine and feline diets^1,2^

**DOI:** 10.1093/tas/txaa133

**Published:** 2020-07-21

**Authors:** Lauren M Reilly, Patrick C von Schaumburg, Jolene M Hoke, Gary M Davenport, Pamela L Utterback, Carl M Parsons, Maria R C de Godoy

**Affiliations:** 1 Department of Animal Sciences, University of Illinois, Urbana, IL; 2 Archer Daniels Midland Company, Decatur, IL

**Keywords:** cat, cecectomized rooster, dog, protein concentrates, protein quality, yeast

## Abstract

Increased consumer interest in high-quality and novel protein sources has driven the demand for the inclusion of protein-rich ingredients in companion animal diets. Novel protein concentrates, with protein contents of at least 50%, have been used to satisfy these consumer demands. However, minimal information is available regarding the macronutrient composition and protein quality of these ingredients that is needed for proper formulation of pet foods. Therefore, the objectives of this study were to determine the macronutrient and amino acid compositions, standardized amino acid digestibility according to the precision-fed rooster assay, and protein quality using digestible indispensable amino acid score (DIAAS like) of pea protein (PP), potato protein (POP), faba bean protein (FBP), soy protein concentrate (SPC), and dried yeast (DY). Precision-fed rooster assays were conducted using cecectomized roosters to calculate standardized amino acid digestibility and true metabolizable energy corrected for nitrogen (TMEn). For all five protein concentrates, all essential amino acids were highly digestible (88.0% to 96.3%, dry matter basis) with differences (*P* < 0.05) in only lysine, methionine, and tryptophan digestibilities. The TMEn values were highest for POP (4.22 kcal/g) and DY (3.61 kcal/g). The DIAAS-like values for adult dogs indicated that methionine was the first-limiting amino acid in all protein concentrates except POP, where the first-limiting amino acid was tryptophan. Using Association of American Feed Control Officials (AAFCO)-recommended values for adult cats, DIAAS-like values for methionine were lowest (*P* < 0.05) for FBP at 81.5%, with all other amino acids for all protein concentrates over 100%. The National Research Council (NRC)-recommended allowances for adult cats indicated that DIAAS-like methionine values for PP (92.7%) and FBP (73.8%) were significantly lower (*P* < 0.05) with these being the first-limiting amino acids, with the remaining amino acids above 100% for the other protein concentrates. The protein quality and high essential amino acid digestibility of these protein concentrates indicate that they would be viable protein sources in canine and feline diets. However, additional complementary protein sources should be included to meet the requirements of all essential amino acids.

## INTRODUCTION

Recent trends in the pet food industry have been centered on providing high-protein diets that resemble the dietary habits of the wild ancestors of dogs and cats. Although high-protein diets typically contain animal-derived protein that often is demanded by consumers, the sustainability of these diets has been questioned (Raditic et al., 2011; Swanson et al., 2013). The use of plant-based proteins in pet foods is an inexpensive and sustainable alternative to traditional animal protein sources. With 90 million dogs and 94 million cats currently living in the United States, protein-rich ingredients, such as protein concentrates, can be utilized to sustain the production of these high-protein diets (Venturini et al., 2018; APPA, 2019).

Protein concentrates are commonly produced through either solubilization, the process of aggregating protein molecules out of solution, or eliminating nonprotein fractions (Ma, 2004; Garba and Kaur, 2014). The amount of protein present in plant protein concentrates ranges from 50% to 70%, depending on growing and processing conditions (Clapper et al., 2001; Houde et al., 2018). Novel protein concentrates, extracted from legumes, potatoes, and yeast, have not been extensively evaluated, limiting their potential for use in canine and feline diets.

Due to the novelty of these protein concentrates, minimal information is available on their amino acid digestibility and protein quality. Therefore, the objectives of this study were to evaluate soy protein concentrate (SPC), faba bean protein (FBP), pea protein (PP), potato protein (POP), and a dried yeast protein (DY) for macronutrient composition, amino acid profile, standardized amino acid digestibility using the precision-fed cecectomized rooster assay, and protein quality calculated using digestible indispensable amino acid score (DIAAS like). The precision-fed rooster assay has proven to be an accurate model for companion animal nutrition based on its correlation to results from ileal-cannulated dogs (Johnson et al., 1998). Although the use of DIAAS-like scores to evaluate ingredients used in pet foods is only just emerging (Oba et al., 2019; Do et al., 2020), it is the recommended method of evaluating protein quality according to the FAO Expert Consultation (FAO, 2013). It was hypothesized that the amino acids in the select protein concentrates would be highly digestible in the cecectomized rooster model and could be considered high-quality protein sources for use in canine and feline diets. It also was expected that methionine would be the first-limiting amino acid in legume-derived protein ingredients tested herein.

## MATERIALS AND METHODS

All animal protocols used in this study were approved by the Institutional Animal Care and Use Committee at the University of Illinois at Urbana–Champaign. All methods were performed in accordance with the United States Public Health Service Policy on Humane Care and Use of Laboratory Animals.

### Sample Preparation and Chemical Analyses

The fine protein concentrates (Archer Daniels Midland, Decatur, IL) were ground through a 2-mm screen in a Wiley mill (model 4; Thomas Scientific, Swedesboro, NJ) and were analyzed in duplicate for dry matter (DM), ash, and organic matter (OM) according to AOAC (2006; methods 934.01 and 942.05). Crude protein (CP) was calculated from Leco (TruMac N, Leco Corporation, St. Joseph, MI), and total nitrogen values were determined according to AOAC (2006; method 992.15). Gross energy (GE) was measured using bomb calorimetry (Model 6200, Parr Instruments Co., Moline, IL). Acid-hydrolyzed fat (AHF) was used to measure total fat content according to AACC (1983) and Budde (1952). Total dietary fiber (TDF) was measured according to Prosky et al. (1992). Last, a complete amino acid profile was determined for each of the five protein concentrates according to AOAC (2007). A coefficient of variance of 5% was used for all chemical analyses.

### Precision-Fed Rooster Assay

A precision-fed rooster assay was conducted according to Parsons (1985). The assay used 20 cecectomized, single-comb White Leghorn roosters with four roosters per treatment. The roosters were housed in a temperature-controlled room on a 16-h light and 8-h dark cycle. All roosters were housed in cages with wire floors, allowing the excreta to be quantitatively collected. Prior to the trial, all roosters were fasted for 26 h. On the day of the trial, roosters were crop intubated with 30 g of a protein concentrate and corn mixture in a 1:1 ratio. After 48 h, excreta were collected, freeze-dried, and ground to a consistent particle size. The excreta was later analyzed for amino acids (AOAC, 2007) Standardized amino acid digestibility was calculated using average endogenous values calculated from multiple roosters over the course of several years. The standardized amino acid profile was calculated according to Sibbald (1979) using the following equations:

$$\begin{array}{l} \begin{array}{ll} & Step\;1:\;Mixed\;amino\;acid\;digestibility,\;\%\\ & =\;\frac{FAA-EAA+EndAA}{FAA}\times 100\\ & Step\;2:\;Standardized\;amino\;acid\;digestibility,\;\%\\ & =\;\frac{AAD_{c}-(AAD_{c}-AAD_{m})}{FAAratio}\times 100 \end{array} \end{array}$$ (1)

A mixed amino acid digestibility value was calculated for the combination of corn and protein concentrate where FAA is the total amino acids fed; EAA is the total amino acids voided in the excreta; and EndAA is the total endogenous amino acids voided in the excreta of fasted roosters. To correct for the added corn, the standardized amino acid for each protein concentrate was calculated where AAD_c_ is the amino acid digestibility of the corn; AAD_m_ is the mixture amino acid digestibility; and FAA ratio is the ratio of amino acid content (%) of the protein concentrate to the amino acid content (%) of the protein concentrate and added corn.

True metabolizable energy corrected for nitrogen (TMEn) was calculated according to Parsons and Baker (1982) using the following equations:

$$\begin{array}{l} \begin{array}{ll} & Step\;1:\;Mixed\;TMEn,\;kcal/g\\ & =\frac{FE_{fed}-(EE_{fed}+8.22(N_{fed}))+(EE_{fasted}+8.22(N_{fasted}))}{FI}\\ & Step\;2:\;TMEn,\;kcal/g\\ & =\frac{TMEn_{c}-(TMEn_{c}-TMEn_{m})}{0.5} \end{array} \end{array}$$ (2)

In the above equations, a mixed TMEn value was first calculated for the combination of the corn and protein concentrate. In the mixed TMEn equation, FE_fed_ is the GE of the feed (kcal); EE_fed_ and EE_fasted_ are the total voided excreta energy (kcal) by the fed and fasted roosters, respectively; 8.22 is the GE per gram of nitrogen of uric acid, N_fed_ and N_fasted_ are the amount of retained nitrogen (g) in the fed and fasted birds, respectively; and FI is the total feed intake. The added corn was factored out of the final TMEn equation where TMEn_c_ is the TMEn of the added corn, TMEn_m_ is the TMEn of the 1:1 (or 50:50 substitution) of the corn to protein concentrate ingredient, and 0.5 corrects for this ratio of corn to protein concentrate ingredient.

### DIAAS-Like Values

Modified DIAAS-like scores were calculated according to Mathai et al. (2017) to determine the protein quality using the standardized amino acid digestibility calculated from the precision-fed cecectomized rooster assay. Reference protein patterns (mg/g) were calculated by determining how much of each indispensable amino acid (mg) was present in 1 g of protein based on Association of American Feed Control Officials (AAFCO)-recommended values or the National Research Council (NRC)-recommended allowances for adult dogs and cats at maintenance. Similarly, the amount of each indispensable amino acid (mg) present in 1 g of protein in each of the five protein concentrates was calculated. The DIAAS-like score could then be calculated using the following equation:

$$\begin{array}{ll} & DIAAS\text{-}like,\;\%\\ & =\frac{mg\;of\;digestible\;AA\;in\;1\;g\;\;of\;dietary\;protein\;}{mg\;of\;same\;AA\;in\;1\;g\;of\;reference\;protein}\;\\ & \times 100 \end{array}$$ (3)

For each protein concentrate, a DIAAS-reference score of 100% or above is considered to be high quality, scores below 100% but higher than 50% are considered to be of moderate quality, and scores lower than 50% are considered to be insufficient as a primary source for the respective amino acid (Mathai et al., 2017). The amino acid with the lowest DIAAS-reference score (the DIAAS-like score) determines the overall protein quality and the first-limiting amino acid. For ingredients that have over 100% DIAAS-like scores for all amino acids, no amino acid is limiting.

### Statistical Analyses

All data were analyzed in SAS (SAS Institute Inc., version 9.4, Cary, NC) using the Mixed Models procedure. The model for the precision-fed rooster assay was performed with a fixed effect of treatment and a random effect of rooster. Differences among treatments were reported using a Fisher-protected least significant difference test with a Tukey adjustment to control for a type-1 experiment-wise error. Differences among treatments were considered statistically significant using a probability of *P* < 0.05. The SEM were reported based on the Mixed Models procedure in SAS.

## RESULTS AND DISCUSSION

### Chemical Composition of Protein Concentrates

The chemical composition of protein concentrates (Table 1) was highly variable. Pea protein concentrate is produced through alcohol extraction and drying processes (Tömösközi et al., 2001). Potato protein concentrate is developed through spray-drying after either polyelectrolyte coagulation, ultrafiltration, or cryocentration of potato juice that contains 30% to 41% protein (Wojnowska et al., 1981). Faba beans are dehulled, milled, and air classified into starch and protein fractions (Sosulski and McCurdy, 1987). Soy protein concentrate is produced from defatted soy flakes and removal of all soluble carbohydrates. Soy protein concentrates then can be texturized or ground to a specific size to aid in the final product characteristics (Peisker, 2001). Last, yeast (*Saccharomyces cerevisiae*) products are single-celled fungi that are an abundant by-product of the brewing industry (Rose, 1960; Ferreira et al., 2010). Yeast protein extraction often is laborious due to the need for the enzymatic removal of the cell wall and difficult solubilizations caused by the inability of solvents to efficiently extract all protein fractions (Horvath and Riezman, 1994; von der Haar, 2007). However, recent methods have focused on alkaline extraction to increase permeability of the yeast cell wall, which increases efficiency of extraction while maintaining the integrity of the functional properties of yeast (Kushnirov, 2000; Zhang et al., 2011). The variation in source of the protein ingredients and their processing methods is reflected in the variation noted in macronutrient composition.

**Table 1.** Macronutrient composition of select plant and yeast protein concentrates

Item	Pea protein	Treatments			
		Potato protein	Faba bean protein	Soy protein concentrate	Dried yeast
Dry matter, %	91.0	90.6	89.7	94.3	92.8
	%, DM^1^ basis				
Organic matter, %	94.1	97.6	94.2	93.4	94.8
Crude protein, %	55.1	80.8	64.6	72.3	52.4
Acid-hydrolyzed fat, %	5.0	3.1	4.1	1.1	15.7
Total dietary fiber, %	22.7	30.6	22.1	31.9	38.3
Gross energy, kcal/g, measured^2^	4.8	5.5	4.9	4.7	5.6

^1^DM = dry matter. ^2^Gross energy was measured using bomb calorimetry.

The DM content of the concentrates ranged from 89.7% in FBP to 94.3% in SPC. The OM content [DM basis (DMB)] was similar among PP, FBP, SPC, and DY with an average of 94.1%. The OM content of POP was slightly higher at 97.6%. A large amount of variation was observed in the CP content (DMB) of the concentrates. The highest CP content was for POP (80.8%), followed by SPC at 72.3%. Although the analyzed samples are from a single harvest, which can be considered a limitation of the study, the chemical composition of these ingredients was consistent with previously reported values. A study evaluating POP concentrates processed using ammonium sulfate precipitation or isoelectric precipitation reported higher CP values of 85.4% and 88.8% (DMB), respectively (Zhang et al., 2017). The CP content of POP is higher than what is typically observed in protein concentrates. In the majority of protein concentrates, CP content ranges from 50% to 70% (Clapper et al., 2001; Houde et al., 2018). Average CP contents of yeast extracts typically range from 73% to 75% (Rakowska et al., 2017). The CP content of SPC was similar to previous literature. One study evaluating legume protein concentrates measured SPC at 72.0% (Hopkins et al., 2019). In the current study, lower CP values were measured in FBP (64.6%), PP (55.1%), and DY (52.4%). One study evaluating protein availability in FBP and PP concentrates reported similar CP contents at 60.9% and 50.0% (DMB), respectively (Carnovale et al., 1988). The CP in DY is similar to previously reported protein analyses of yeast. Brewer’s yeast has been reported to have a CP content of 50.2% (DMB), whereas sugarcane yeast is slightly lower, ranging from 42.5% to 45.5% (Martins et al., 2013).

The DY had the highest AHF value of 15.7%, followed by PP with 5.0% and FBP with 4.1%. Lower values were measured for POP (3.1%) and SPC (1.1%). Because SPC is formed from defatted soy flakes, the AHF content is lower than for the other protein concentrates that retain some of the fat content of the whole plant (Peisker, 2001). Previous studies determined fat values (DMB) to be similar for SPC at 0.99% (Valencia et al., 2008) and 0.9% crude fat (Sosulski and McCurdy, 1987). Variable fat contents have been measured for PP. Valencia et al. (2008) reported PP to have 1.8% ether extract, whereas Sosulski and McCurdy (1987) determined field PP concentrate to have a higher crude fat content of 3.7%. The DY in the current study had a higher AHF value than reported in previous studies. Yeast products have been shown to typically have an average of 6% fat (Rakowska et al., 2017). However, the growth temperature of *S. cerevisiae* has been shown to influence lipid composition (Hunter and Rose, 1971). *Saccharomyces cerevisiae* has been shown to be up to 14.4% (DMB) lipid for yeast cultured at 15 °C and ranging from 12.1% to 13.2% for yeast cultured at 30 °C, possibly due to increased synthesis of unsaturated fatty acids at lower temperatures (Hunter and Rose, 1971). In addition, method of determination is likely to affect the results, as acid hydrolysis prior to ether extraction would more efficiently remove the fat content entrapped in the cell membrane of yeast cell wall. The GE content of the by-products is reflective of both the CP and AHF contents. In DY, the GE content was 5.6 kcal/g and was similar to POP that had a GE content of 5.5 kcal/g.

Unlike protein isolates, protein concentrates retain dietary fiber in the final product (Wang et al., 2004). In terms of TDF, similarities were observed between FBP (22.1%) and PP (22.7%) and between POP (30.6%) and SPC (31.9%). The average TDF content of whole pulses ranges from 20.0% to 25.8% (Chen et al., 2016). Faba beans typically contain 27.5% TDF (Karatas et al., 2017). One study evaluated yellow pea flour to have a TDF value of 19%, with PP fractions having 18% TDF (Muneer et al., 2018). Another study evaluating the inclusion of soy proteins in extruded dog diets measured TDF of SPC at 21.3% (DMB), which is lower than in the current study (Clapper et al., 2001). In the current study, the highest TDF value was measured in DY, with a value of 38.3%.

The complete amino acid profile, represented as a percentage of total amino acids, showed varying concentrations of essential amino acids among the concentrates (Table 2). The legume-based sources, PP, FBP, and SPC had lysine concentrations of 7.98%, 7.06%, and 6.55%, respectively. Legumes and pulses are typically high in lysine and low in sulfur-containing amino acids, methionine and cysteine (Agarwal, 2017; Robinson et al., 2019). Soy protein concentrate was evaluated by Clapper et al. (2001) and showed a lysine content of 6.4% and a methionine content of 0.9% (DMB). The majority of the CP content in legumes and pulses originates from the storage proteins present (i.e., globulin and albumin; Agarwal, 2017). Globulins are low in sulfur-containing amino acids, whereas albumins have a high-lysine content (Semba et al., 2016). Higher lysine and methionine contents of an FBP fraction have been reported to be 6.4% and 0.8%, respectively (Sosulski and McCurdy, 1987). In the current study, the methionine content of FBP was 0.78%. Similar to legume and pulse ingredients, POP has been shown to be high in lysine but limiting in sulfur-containing amino acids (Rexen, 1976). In the current study, POP had a similar lysine content of 7.72% to the legume ingredients with a slightly higher methionine content of 2.19%. The methionine content of POP was more similar to the methionine content of DY, which had a value of 2.11%. Additionally, POP and DY had higher leucine concentrations at 9.95% and 11.02%, respectively, compared with the legume ingredients. Zhang et al. (2017) showed POP concentrates with leucine values of 8.2% and 7.8%, for ammonium sulfate precipitation and isoelectric precipitation, respectively. Valine concentrations of these POP concentrates were slightly lower, ranging from 4.83% to 4.78% (Zhang et al., 2017), than the valine concentrations measured in the current study (6.92%).

**Table 2.** Amino acids of select plant and yeast protein concentrates as a percentage of total amino acids

% of total amino acids, DMB^1^	Pea protein	Potato protein	Faba bean protein	Soy protein concentrate	Dried yeast
Essential					
Arginine	9.27	4.88	10.38	7.42	4.48
Histidine	2.60	2.19	2.84	2.69	2.27
Isoleucine	4.87	5.73	4.92	5.01	5.44
Leucine	7.77	9.95	8.13	7.87	11.02
Lysine	7.98	7.72	7.06	6.55	6.04
Methionine	0.93	2.19	0.78	1.47	2.11
Phenylalanine	5.25	6.28	4.78	5.14	5.48
Threonine	3.62	5.39	3.52	3.81	4.32
Tryptophan	0.91	0.88	0.90	1.23	1.04
Valine	5.33	6.92	5.32	5.32	6.26
Nonessential					
Alanine	4.38	4.66	4.32	4.34	7.01
Aspartate	11.98	12.20	11.47	11.55	8.95
Cysteine	1.21	1.48	1.21	1.44	1.33
Glutamate	16.93	10.36	17.34	18.59	14.50
Glycine	4.45	4.84	4.49	4.31	4.44
Proline	4.68	4.99	4.83	5.35	6.24
Serine	3.81	4.09	3.96	4.02	4.04
Tyrosine	3.34	5.07	3.37	3.48	4.08
Total amino acids, %	52.75	84.29	58.78	72.19	49.81

^1^DMB = dry matter basis.

### Precision-Fed Rooster Assay

The standardized amino acid digestibility (Table 3) was calculated from the precision-fed cecectomized rooster excreta. The use of the precision-fed cecectomized rooster assay has been demonstrated to be an acceptable model for estimating canine in vivo amino acid digestibility. The cecectomized rooster model has demonstrated similar results to ileal-cannulated dogs (Johnson et al., 1998). The accuracy of using cecectomized roosters as a model in feline nutrition has not been determined. However, it is generally accepted that amino acid digestibility differences are negligible between dogs and cats when amino acids are over 90% digestible (Kendall et al., 1982; Kerr et al., 2013). Protein concentrates were mixed in a 1:1 ratio with raw, ground corn in order to limit adherence to the sides of the tube during crop intubation. The addition of corn ensured that all protein concentrates were quantitatively deposited into the crop of the rooster. Corn is a low-protein ingredient that accounts for minimal amino acids in the mixture. Protein-free ingredients, such as glucose or cornstarch, exacerbate the adherence of the mixture to the tube and, therefore, could not be used. The amino acids and endogenous values for the corn used were analyzed from a single harvest and could therefore be factored out of the equation to provide the standardized digestibility of the protein concentrate.

**Table 3.** Standardized amino acid digestibilities of plant and yeast protein concentrates calculated using the precision-fed rooster assay^1^

%, DM^2^ basis	Treatment					
Essential amino acid	Pea protein	Potato protein	Faba bean protein	Soy protein concentrate	Dried yeast	SEM
Arginine	96.3	96.1	94.6	95.9	95.2	0.909
Histidine	93.7	93.6	91.1	94.4	91.7	1.409
Isoleucine	92.4	94.8	92.8	94.1	92.2	1.063
Leucine	93.2	95.5	93.3	93.5	94.1	1.062
Lysine	93.1^a^	93.6^a^	89.8^ab^	93.1^a^	88.4^b^	1.302
Methionine	88.5^bc^	95.4^a^	88.0^c^	93.1^ab^	93.6^a^	1.582
Phenylalanine	93.3	95.3	93.3	94.4	93.3	1.023
Threonine	92.5	94.1	90.5	92.0	86.1	1.835
Tryptophan	93.4^abc^	94.6^ab^	93.2^bc^	96.3^a^	91.7^c^	0.972
Valine	91.8	94.6	91.4	93.1	91.8	1.357

^a,b^Means within a row with different superscript letters are different (*P* < 0.05). ^1^ *n* = 4 cecectomized roosters per treatment. ^2^DM = dry matter.

Significant differences were observed only in digestibility of lysine, methionine, and tryptophan for the concentrates. The standardized lysine digestibility was lowest (*P* < 0.05) for DY with a value of 88.4% but was not significantly different (*P* > 0.05) than FBP (89.8%). The lysine digestibilities for PP, POP, and SPC were 93.1%, 93.6%, and 93.1%, respectively. The standardized methionine digestibility was lowest (*P* < 0.05) for PP (88.5%) and FBP (88.0%). The tryptophan digestibility was highest (*P* < 0.05) for SPC (96.3%), but not significantly different (*P* > 0.05) than PP (93.4%) or POP (94.6%). All essential amino acids were highly digestible with values ranging from 88.0% to 96.3% for all essential amino acids. However, it should be noted that further pet food processing methods, such as extrusion, can alter nutrient digestibility and amino acid bioavailability (Tran et al., 2008; van Rooijen et al., 2013). Cotten et al. (2016) determined the standardized amino acid digestibility of POP and soy protein using ileal-cannulated pigs. Potato protein digestibility ranged from 94.3% to 96.8% for all essential amino acids. Soy protein ranged from 88.3% to 96.5% for all essential amino acids (Cotten et al., 2016). Similarly, Oliveira and Stein (2016) showed SPC ranging from 88.7% to 95.5% for all essential amino acids. The standardized ileal digestibility of yeast-derived protein has been measured in ileal-cannulated weaned pigs (Moehn et al., 2010). The standardized ileal lysine digestibility was lower than that measured in the present study at 79.5% (Moehn et al., 2010).

**Figure 1.**
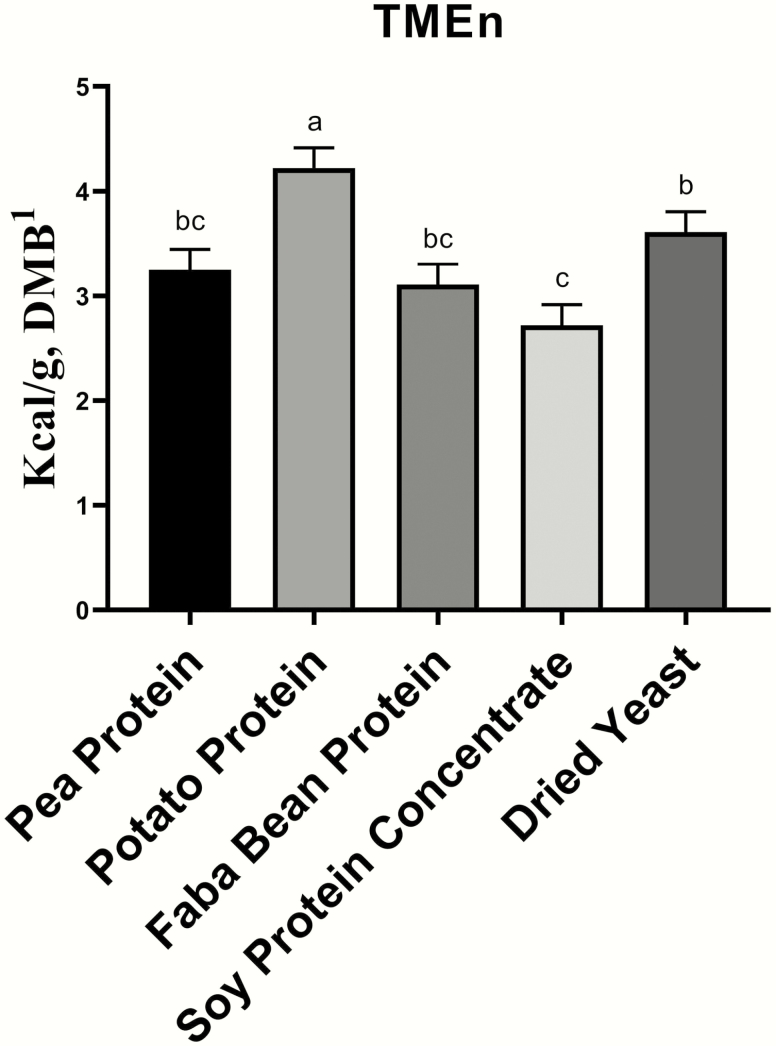
The true metabolizable energy corrected for nitrogen values calculated for each of the protein concentrates. ^1^DMB = dry matter basis

The TMEn values were calculated for each of the protein concentrates (Fig. 1). The TMEn values were calculated in order to account for endogenous energy losses and higher nitrogen losses in fasted birds when compared with fed birds. The correction for these losses provides a more accurate measure of true metabolizable energy (Parsons et al., 1982). An accurate measure of metabolizable energy is needed in order for these ingredients to be included in pet formulations while meeting specific energy targets. The TMEn of the corn was analyzed from the same harvest that was used in the trial and could be factored out of the equation. For the protein concentrates, POP had the highest (*P* < 0.05) TMEn value (DMB) of 4.22 kcal/g. The lowest (*P* < 0.05) TMEn value was measured for SPC, with a value of 2.72 kcal/g. The TMEn of SPC was not significantly different (*P* > 0.05) than PP or FBP, which have TMEn values of 3.25 and 3.11 kcal/g, respectively. These values are similar to the TMEn value reported by Parsons et al. (1982) for dehulled soybean meal with a TMEn value of 2.95 kcal/g (DMB). Traditional protein sources used in the pet food industry, such as chicken-based ingredients, have been reported by Oba et al. (2019). Chicken meal was reported to have a TMEn value of 3.72 kcal/g (DMB), whereas raw, steamed, and retorted chicken products had higher TMEn values, with an average of 5.59 kcal/g (Oba et al., 2019). The differences observed in the study by Oba et al. (2019) is reflective of how processing conditions and variation in the composition of animal-derived protein sources can influence energy content of ingredients, which is also observed, to a lesser extent, among the protein concentrates tested in the current study.

### DIAAS-Like Values

Traditional DIAAS values are calculated using standardized amino acid digestibility determined from ileal-cannulated pigs. For these values, the reference protein pattern used is the estimated average requirement of 2- to 5-yr-old children (Marinangeli and House, 2017). In the current study, the DIAAS-like values were calculated using standardized amino acid digestibility from cecectomized roosters. Reference protein patterns were determined from AAFCO-recommended values and NRC-recommended allowances of adult dogs (Tables 4 and 5) and cats (Tables 6 and 7) at maintenance. The previous FAO-established method of determining protein quality, known as protein digestibility-corrected amino acid scores (PDCAAS), truncated scores at 100%, causing the underestimation of high-quality protein sources (FAO/WHO, 1991). The use of DIAAS scores avoids the faults of the PDCAAS system and allows for the accurate evaluation of high-quality proteins (FAO, 2013; Mathai et al., 2017).

**Table 4.** Digestible indispensable amino acid score (DIAAS)-like^1^ values for select plant and yeast protein concentrates compared with AAFCO-recommended values for adult dogs at maintenance

%, DM^2^ basis	Treatment					
Essential amino acid	Pea protein	Potato protein	Faba bean protein	Soy protein concentrate	Dried yeast	SEM
Arginine	300.6^b^	171.7^d^	314.1^a^	250.0^c^	142.5^e^	1.964
Histidine	218.6^b^	198.7^c^	220.8^b^	237.5^a^	185.3^d^	3.065
Isoleucine	204.3^c^	268.5^a^	196.7^c^	223.3^b^	226.5^b^	2.613
Leucine	183.6^c^	262.6^a^	182.5^c^	194.6^b^	260.7^a^	2.497
Lysine	202.8^b^	214.9^a^	164.4^c^	173.6^c^	144.6^d^	2.497
Methionine	42.7^d^	118.6^a^	34.0^e^	74.1^c^	99.9^b^	1.479
Phenylalanine	186.9^b^	248.7^a^	161.6^c^	193.1^b^	193.6^b^	2.132
Threonine	120.4^cd^	198.3^a^	108.8^d^	131.4^bc^	132.5^b^	2.767
Tryptophan	91.6^d^	97.5^c^	86.1^e^	133.5^a^	102.4^b^	1.109
Valine	171.4^cd^	249.9^a^	162.1^d^	181.1^c^	200.0^b^	2.821

^a–e^Means within a row with different superscript letters are different (*P* < 0.05). ^1^ DIAAS-like (%) = [(mg of digestible indispensable amino acid in 1 g of dietary protein)/mg of same indispensable AA in 1 g of reference protein)] × 100. ^2^DM = dry matter.

**Table 5.** Digestible indispensable amino acid score (DIAAS)-like^1^ values for select plant and yeast protein concentrates compared with NRC-recommended allowances for adult dogs at maintenance

%, DM^2^ basis	Treatment					SEM
Essential amino acid	Pea protein	Potato protein	Faba bean protein	Soy protein concentrate	Dried yeast	
Arginine	244.3^b^	171.1^d^	255.3^a^	203.2^c^	115.8^e^	1.691
Histidine	122.7^b^	111.6^c^	124.0^b^	133.4^a^	104.1^d^	1.721
Isoleucine	113.5^c^	149.1^a^	109.3^c^	124.0^b^	125.8^b^	1.451
Leucine	102.0^c^	145.9^a^	101.4^c^	108.1^b^	144.8^a^	1.387
Lysine	203.4^b^	215.6^a^	164.9^c^	174.1^c^	145.1^d^	2.506
Methionine	23.9^d^	66.3^a^	18.9^e^	41.4^c^	55.9^b^	0.827
Phenylalanine	104.3^b^	138.8^a^	90.2^c^	107.7^b^	108.0^b^	1.189
Threonine	74.6^cd^	123.0^a^	67.5^d^	81.5^bc^	82.2^b^	1.717
Tryptophan	58.2^d^	61.9^c^	54.7^e^	84.8^a^	65.0^b^	0.647
Valine	95.6^cd^	139.4^a^	90.4^d^	101.0^c^	111.6^b^	1.573

^a–e^Means within a row with different superscript letters are different (*P* < 0.05). ^1^DIAAS like, % = [(mg of digestible indispensable amino acid in 1 g of dietary protein)/mg of same indispensable AA in 1 g of reference protein)] × 100. ^2^DM = dry matter.

**Table 6.** Digestible indispensable amino acid score (DIAAS)-like^1^ values for select plant and yeast protein concentrates compared with AAFCO-recommended values for adult cats at maintenance

%, DM^2^ basis	Treatment					
Essential amino acid	Pea protein	Potato protein	Faba bean protein	Soy protein concentrate	Dried yeast	SEM
Arginine	213.7^b^	122.1^d^	223.3^a^	177.8^c^	101.3^e^	1.396
Histidine	194.3^b^	176.7^c^	196.3^b^	211.2^a^	164.8^d^	2.726
Isoleucine	215.7^c^	283.4^a^	207.6^c^	235.7^b^	239.1^b^	2.757
Leucine	145.5^c^	208.0^a^	144.6^c^	154.1^b^	206.5^a^	1.978
Lysine	222.5^b^	235.8^a^	180.3^c^	190.5^c^	158.7^d^	2.741
Methionine	102.4^d^	284.3^a^	81.5^e^	177.6^c^	239.8^b^	3.547
Phenylalanine	290.7^b^	386.7^a^	251.3^c^	300.2^b^	300.9^b^	3.314
Threonine	114.0^c^	187.9^a^	103.1^d^	124.5^b^	125.6^b^	2.621
Tryptophan	132.4^d^	140.9^c^	124.4^e^	192.9^a^	148.0^b^	1.473
Valine	196.4^cd^	286.5^a^	185.8^d^	207.5^c^	229.2^b^	3.233

^a–e^Means within a row with different superscript letters are different (*P* < 0.05). ^1^DIAAS-like, % = [(mg of digestible indispensable amino acid in 1 g of dietary protein)/mg of same indispensable AA in 1 g of reference protein)] × 100. ^2^DM = dry matter.

**Table 7.** Digestible indispensable amino acid score (DIAAS)-like^1^ values for select plant and yeast protein concentrates compared with NRC-recommended allowances for adult cats at maintenance

%, DM^2^ basis	Treatment					
Essential amino acid	Pea protein	Potato protein	Faba bean protein	Soy protein concentrate	Dried yeast	SEM
Arginine	222.1^b^	126.9^d^	232.0^a^	184.7^c^	105.3^e^	1.451
Histidine	179.4^b^	163.1^c^	181.2^b^	194.9^a^	152.1^d^	2.515
Isoleucine	200.6^c^	263.6^a^	193.1^c^	219.2^b^	222.4^b^	2.565
Leucine	136.0^c^	194.5^a^	135.2^c^	144.1^b^	193.1^a^	1.850
Lysine	418.7^b^	443.8^a^	339.5^c^	358.5^c^	298.6^d^	5.161
Methionine	92.7^d^	257.2^a^	73.8^e^	160.7^c^	216.9^b^	3.210
Phenylalanine	234.7^b^	312.2^a^	202.9^c^	242.4^b^	243.0^b^	2.676
Threonine	123.4^cd^	203.4^a^	111.6^d^	134.7^bc^	135.9^b^	2.838
Tryptophan	125.3^d^	133.4^c^	117.7^e^	182.5^a^	140.0^b^	1.394
Valine	183.7^cd^	267.9^a^	173.7^d^	194.1^c^	214.4^b^	3.023

^a–e^Means within a row with different superscript letters are different (*P* < 0.05). ^1^DIAAS like, % = [(mg of digestible indispensable amino acid in 1 g of dietary protein)/mg of same indispensable AA in 1 g of reference protein)] × 100. ^2^DM = dry matter.

Using AAFCO (2018)-recommended values for adult dogs at maintenance as the reference protein, most essential amino acids had DIAAS-reference values over 100%. Moderate-quality DIAAS-like methionine scores were calculated for SPC (74.1%) and DY (99.9%). Low-quality DIAAS-like methionine scores were calculated for PP (42.7%) and FBP (34.0%). The first-limiting amino acid in these four concentrates was methionine. In POP, DIAAS-reference scores were over 100% (ranging from 118.6% to 268.5%) for all essential amino acids with the exception of its first-limiting amino acid (tryptophan) that had a DIAAS-like score of 97.5%. The same pattern was observed when DIAAS-like scores were calculated using NRC (2006)-recommended allowances for adult dogs at maintenance. However, moderate-quality scores were calculated for phenylalanine in FBP, threonine in PP, FBP, SPC, and DY, tryptophan for all five concentrates, and valine for PP and FBP. These lowered DIAAS-reference values compared with AAFCO-recommended allowances are a result of higher amino acid requirements set by the NRC.

Minimal data had been reported for DIAAS-like values for use in canine and feline nutrition. Recently, DIAAS-like values have been used to evaluate novel protein sources (Do et al., 2020) and traditional protein sources (Oba et al., 2019) used in companion animal diets. The determination of DIAAS-like scores for both traditional and novel protein sources helps to identify potential complementary protein sources that could be included in diet formulations to meet the requirements for all essential amino acids. Do et al. (2020) evaluated DIAAS-like scores for black soldier fly larvae at various ages for both growing puppies and adult dogs. Compared with AAFCO-recommended values for adult dogs, the first-limiting amino acid was methionine for all larvae ages with DIAAS-like scores ranging from 73% to 93%. These results resemble those of the current study, as the first-limiting amino acid for all protein concentrates, with the exception of POP, was methionine. Chicken-based ingredients were evaluated by Oba et al. (2019), which allows for comparison of the novel plant-based protein sources evaluated in the current study to traditional animal proteins. Oba et al. (2019) determined the DIAAS-like scores for raw chicken, retorted chicken, steamed chicken, and chicken meal. When using the AAFCO-recommended values for adult dogs as the reference protein, methionine was the first-limiting amino acid for chicken meal, whereas tryptophan was the first-limiting amino acid for the remaining chicken ingredients (Oba et al., 2019). Because methionine was the first-limiting amino acid in the black soldier fly larvae (Do et al., 2020) and chicken meal (Oba et al., 2019), these ingredients could not be used as complementary protein sources for the legume-based or yeast ingredients evaluated in the current study for adult dogs.

In the current study, the DIAAS-reference scores calculated from AAFCO-recommended values for adult cats at maintenance were over 100% for all essential amino acids for PP, POP, SPC, and DY. Because all values were over 100% for these concentrates, no first-limiting amino acid can be identified. For FBP, methionine was the first-limiting amino acid with a DIAAS-like score of 81.5%. All other essential amino acids had DIAAS-reference scores over 100% for FBP. The NRC-recommended allowances showed DIAAS-reference scores over 100% for POP, SPC, and DY. For PP and FBP, the first-limiting amino acid was methionine with values of 92.7% and 73.8%, respectively. Do et al. (2020) reported that no limiting amino acid could be identified for any of the black soldier fly larvae ages for adult cats. Therefore, black soldier fly larvae could be included as a complementary novel protein source for the plant- or yeast-concentrated proteins analyzed in the current study. Oba et al. (2019) determined threonine to be the first-limiting amino acid in the chicken ingredients when compared with AAFCO-recommended values (91.5%) and NRC-recommended allowances (98.8%) for adult cats at maintenance.

Hughes et al. (2011) evaluated the protein quality of SPC and soy protein isolates using PDCAAS. Both the SPC and the soy protein isolate had truncated PDCAAS values of 1.0. As mentioned previously, the truncation of these values underestimates the nutritive value of high-quality proteins, as the SPC in the current study had DIAAS-like scores over 100% for all amino acids, with the exception of lysine in adult dogs. The protein quality of PP and POP has been evaluated using the protein efficiency ratio (PER) in chicks (Donadelli et al., 2019). Compared with the PER value of spray-dried egg, which has a value of 5.2, POP isolate had a value of 3.6, whereas PP isolate had a lower value of 1.9 (Donadelli et al., 2019). The higher PER value of POP compared with PP can be attributed to the high amino acid digestibility, as was observed in the current study.

## CONCLUSIONS

The use of protein concentrates is highly beneficial in canine and feline diets. These energy-dense ingredients would meet nutritional requirements of dogs and cats while meeting consumer demand for novel and high-quality protein sources. The inclusion of these ingredients would improve sustainability and reduce manufacturing costs of pet food compared with the use of animal-derived proteins. All amino acids were highly digestible according to the precision-fed cecectomized rooster assay, which was reflected in the determination of DIAAS-like scores. The DIAAS-like method of evaluating protein quality was used in this study to accurately evaluate the protein quality of the plant and yeast ingredients. Through the determination of the DIAAS-like scores, methionine was identified as the first-limiting amino acid for most of the analyzed ingredients according to both canine and feline requirements. These protein concentrates would need to be complemented with ingredients with high sulfur-containing amino acids, such as cereal grains, to meet requirements. However, future studies will be needed in order to determine the effects of the inclusion of these ingredients in canine and feline diets on macronutrient digestibility, acceptability, and fecal characteristics of dogs and cats.
